# ART TO LIFE: THE IMPACT OF AN EXPERIENTIAL ARTS PROGRAM ON ENGAGEMENT IN PERSONS LIVING WITH DEMENTIAS

**DOI:** 10.1093/geroni/igz038.3250

**Published:** 2019-11-08

**Authors:** Candice Reel

**Affiliations:** The University of Alabama, Tuscaloosa, Alabama, United States

## Abstract

The Art to Life (ATL) program aims to improve the quality of life for persons living with dementia (PWD) through art therapy, intergenerational contact with college students, and life story preservation within an adult day service. This poster will present the results of an ongoing program evaluation to determine the effects of the intervention on PWDs’ engagement in (1) communication with others and (2) art/creative activity. A two-member analysis team independently coded ethnographic field notes utilizing operational definitions of PWDs’ observed behavior during momentary time sampling, and recording events of communication and art engagement using the modified ENGAGE measure (Hartmann et. al, 2017). Results across sessions (N=97) reveal communication engagement (M=28.30, SD=13.36) significantly exceeded art engagement (M=9.86, SD=5.56), t (96)=20.85, p=0.001). These results suggest that engagement in reminiscence via intergenerational contact is a fundamental feature in comparison to art and creative activity within the ATL program. Exploratory qualitative content analysis of ethnographic field notes by a three-member coding team identified two key themes within the communication engagements. These emergent themes included validation of personhood and reminiscence of family ties. More studies are needed to determine if the scope and reach of intergenerational interventions may be increased through the nationwide translation of the ATL program.

